# Editorial: Adipose tissue and adipokines: their roles in human reproduction

**DOI:** 10.3389/fendo.2024.1497744

**Published:** 2024-10-08

**Authors:** Mayank Choubey, Nikolaos Nikolettos

**Affiliations:** ^1^ Department of Foundations of Medicine, Diabetes and Obesity Research Center, New York University (NYU) Grossman Long Island School of Medicine, Mineola, NY, United States; ^2^ Medical School, Democritus University of Thrace, Alexandroupolis, Thrace, Greece

**Keywords:** obesity, adipose tissue, adipokines, adiponectin, human reproduction, male infertility, fertility, metabolic diseases

Our comprehension of the function of adipose tissue has progressed from perceiving it merely as an energy storage organ to recognizing it as an active endocrine organ (1). Although factors such as genetics, sexual characteristics, and sex hormones can affect the quantity and distribution of adipose tissue, it’s important to note that white fat also exert a reciprocal effect on both male and female reproduction (2). The intricate interplay between adipose-derived hormones “adipokines” and human reproductive functions has garnered significant attention in recent years (3). This intricate relationship has been highlighted in our four resourceful manuscripts published in our Research Topic in Frontiers in Endocrinology, focusing on “*Adipose tissue and adipokines: their roles in human reproduction*”. This editorial summarizes the research findings of these studies, highlight their contributions, and outline future avenues for research.

Obesity plays a sophisticated and diversified role in the biology of female reproductive health (Zheng et al.). The first manuscript, “*Obesity and its impact on female reproductive health: unraveling the connections*” (Zheng et al.), explores the multifaceted impact of obesity on female reproductive health. The study comprehensively reviews how excess body fat influences various reproductive parameters, including menstrual cycles, fertility, and pregnancy outcomes. It underscores the need for targeted interventions to mitigate the adverse effects of obesity on women’s reproductive health.

**Figure 1:**
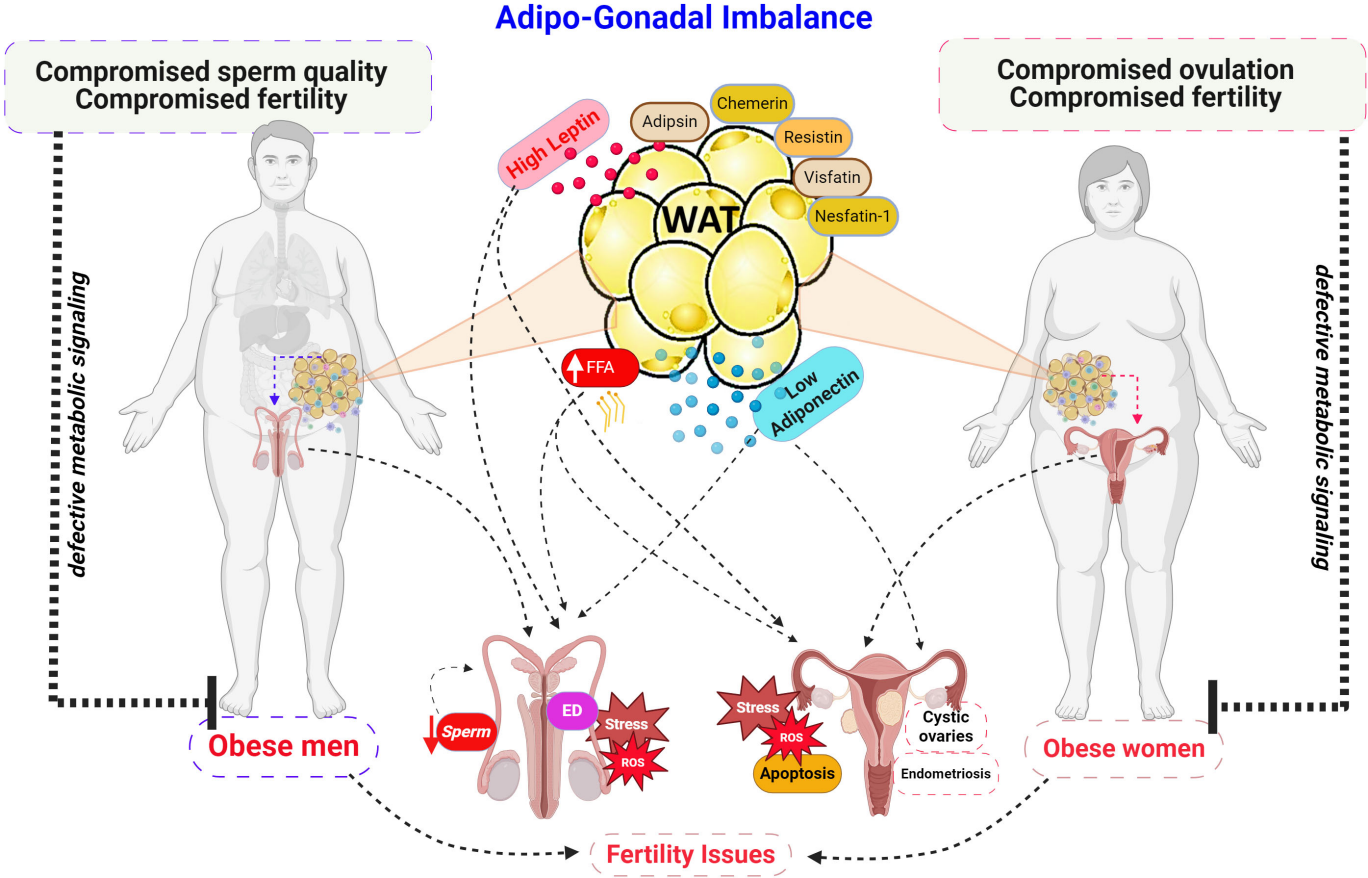
Adipo-Gonadal Imbalance in Adipose-tissue hypertrophy. Schematic representation of the detrimental effects of obesity on male and female reproductive function. Increased adiposity leads to elevated levels of leptin while adiponectin secretion is reduced. These hormonal imbalances, along with increased free fatty acid levels (FFA), contribute to defective metabolic signaling, ultimately impairing sperm quality and erectile dysfunction (ED) in men, disrupting ovulation, and promoting ovarian dysfunction in women. ROS, reactive oxygen species. Created with BioRender.com.

The role of contrary adipokines, specifically leptin and adiponectin, in female reproductive health is a burgeoning research field (**Figure 1**) (2, 4). In “*The association between leptin, adiponectin levels and the ovarian reserve in women of reproductive age*” (Nikolettos et al.), the authors investigate the roles of leptin and adiponectin—two key adipokines—in determining ovarian reserve. Their findings reveal a significant relationship between these adipokines and ovarian function, suggesting that hormonal and metabolic changes may influence female fertility by affecting ovarian reserve.

Shifting the focus to male reproductive health (5), the third manuscript, “*Male infertility risk and plasma lipidome: a Mendelian randomization study*” (Yang Y. et al.) provides a novel perspective. Through Mendelian randomization, the study examines the association between plasma lipid profiles and the risk of male infertility. This research highlights how lipidomic alterations can impact male reproductive health and opens new avenues for potential therapeutic interventions.

The final manuscript in our Research Topic, titled “*Genetic association of lipids and lipid-lowering drug target genes with Endometrial carcinoma: a drug target Mendelian randomization study*” (Yang Z. et al.), delves into the genetic interplay between lipids, lipid-lowering drugs, and endometrial carcinoma. This research employs Mendelian randomization to identify genetic factors that may contribute to the risk of endometrial cancer, offering insights into the role of lipid metabolism in the development of this disease (6).

Collectively, these studies enrich our understanding of how adipose tissue and adipokines impact reproductive health across genders. They offer valuable insights into the mechanisms underlying reproductive disorders and emphasize the importance of integrating metabolic and hormonal factors into reproductive health research.

As we move forward, several research directions emerge. Future studies should explore the potential of targeting adipokines as therapeutic interventions for reproductive disorders. Additionally, the application of multiomics approaches could provide a comprehensive understanding of the complex interactions between adipose tissue, metabolic dysfunction, and reproductive outcomes (7). Further research is needed to elucidate the exact molecular pathways involved in these processes.

In conclusion, this Research Topic has significantly advanced our knowledge of the complex interactions between adipose tissue, adipokines, and human reproduction. We extend our gratitude to all the contributors for their valuable research and look forward to future discoveries in this vital area of study.
